# Report of the Committee on Minimum Standards for Admission to Medical Colleges

**Published:** 1899-08

**Authors:** N. R. Coleman, Augustus Korndorfer, T. J. Happel, Gardner T. Swarts, William R. Tipton

**Affiliations:** Chairman; Ohio; Pennsylvania; Tennessee; Rhode Island; New Mexico


### Society Proceedings.

### REPORT OF THE COMMITTEE ON MINIMUM STANDARDS FOR ADMISSION TO MEDICAL COLLEGES.

At the last annual meeting of the National Confederation of State Medical Examining and Licensing Boards, held at Columbus, O., June 5, 1899, the following report was submitted by a special committee that had considered the subject for three years:

*Mr. President and Gentlemen of the Confederation:*

Your committee, appointed by the President of the National Confederation of State Medical Examining and Licensing Boards, have labored assiduously to inform themselves of the true status of the requirements now in force in the several states, and to obtain the views of members of boards upon the same, and now desire to submit the following report as the result of their labors :

In December, 1897, a letter was prepared and sent to the boards of the several states and territories, for the purpose of ascertaining, as far as possible, the branches in which they believed students should be examined for admission to medical colleges, and the extent of such examinations. Letters were also sent to a number of practical and well-known educators. A large number of replies, embodying very valuable suggestions, were received. Some of these had been acted on in a formal way by boards, others were over the signatures of the secretaries. A large majority of them favored a high standard —much higher than would be possible or even practicable at the present time. By the aid of such information, we hope the committee may be enabled to make a report that may prove in a degree acceptable. We desire to extend our sincere thanks to all who have responded so courteously to our request for information.

In the letter sent to the boards we also requested that they furnish all the information upon this subject embodied in their laws and in the rulings of their respective boards. The commissioners and superintendents of public instruction of a large number of the states were written to, requesting them to forward to the committee the official reports of their departments. The object in obtaining this latter information was to determine, if possible, what class of schools of the higher grade existed in the greatest number in the several states.

In the preliminary report made to the Confederation at Philadelphia, attention was called to the discrepancy in the requirements of the high schools in the several states. Therefore, it is unnecessary to enter into the subject more fully, as the statements then set forth have been verified by more recent research. However, we may be pardoned for inviting attention to the great disparity that now exists in the requirements made by the Association of American Medical Colleges, by the Southern Medical College Association, by the National Confederation of Eclectic Medical Colleges, by the Inter-Collegiate Committee of the American Institute of Homeopathy, by Physio-Medical Colleges, by the states whose minimum requirement is regulated by law, and by those states in which state boards of medical examiners are empowered to adopt rules regulating such requirements. In comparing these we will first call your attention to the

### ASSOCIATION OF AMERICAN MEDICAL COLLEGES.

This association requires an examination in English, arithmetic, algebra, physics and Latin, the minimum of which shall be as follows :

"ARTICLE III.—EXTENT OF THE EXAMINATION.

1. English—A composition on some subject of general interest. This composition must be written by the student at the time of the examination and should contain at least 200 words. It should be criticised in relation to thought, construction, punctuation, spelling and handwriting.

2. Arithmetic—Such questions as will show a thorough knowledge of common and decimal fractions, compound numbers, and ratio and proportion.

3. Algebra—Such questions as will bring out the student's knowledge of the fundamental operations, factoring and simple quadratic equations.

4. Physics—Such questions as will discover the student's understanding of the elements of mechanics, hydrostatics, hydraulics, optics and acoustics.

5. Latin—An examination upon such elementary work as the student may offer, showing a familiarity usually attained by one year of study; for example, the reading of the first fifteen chapters of Cesar's commentaries, and the translation into Latin of easy English sentences involving the same vocabulary.

Sec. 2. In place of this examination, or any part of it, colleges, members of this association, are at liberty to recognise the official certificates of reputable literary and scientific colleges, academies, high schools, and normal schools, and also the medical student's certificate, issued by any state examining board covering the work of the foregoing entrance examination.

Sec. 3. Colleges, members of this association, may allow students *who fail in one or more branches* in this entrance examination the privilege of entering the first-year course, but such students shall not be allowed to begin the second course until the entrance requirements are satisfied.

Sec. 6. Colleges, members of this association, are free to give to students who have met the entrance requirements of the association additional *credit for time* on the four years' course as follows : (*a*) To students having the A. B., B. S., or equivalent *degree* from reputable literary colleges, one year of time. (*b*) To graduates and students of colleges, of *homeopathic* or *eclectic* medicine, as many years as they have attended those colleges, provided they have met the previous requirements of the association and they pass an examination in materia medica and therapeutics. (*c*) To graduates of reputable colleges of *dentistry*, *pharmacy* and *veterinary* medicine, one year of time."

THE SOUTHERN MEDICAL COLLEGE ASSOCIATION

requires that every student applying for matriculation must possess the following qualifications: "He must hold a certificate as the pupil, of some known, reputable physician, showing his moral character and general fitness to enter upon the study of medicine. He must possess a diploma of graduation from some literary or scientific institution of learning, or a certificate from some legally constituted high school, general superintendent of state education, or superintendent of some county board of public education, attesting the fact that he is possessed of at least the educational attainments required of second grade teachers of public schools; provided, however, that if a student so applying is unable to furnish the above and foregoing evidence of literary qualifications, he may be permitted to matriculate and receive medical instruction as other students, qualify himself in the required literary departments, and stand his required examination, as above specified, prior to offering himself for a second course of lectures. The foregoing certificate of educational qualifications attested by the dean of the medical college attended, together with a set of tickets showing that the holder has attended one full course of medical lectures, shall be essential to attendance upon a second course of lectures in any college belonging to the Southern Medical College Association."

THE INTER-COLLEGIATE COMMITTEE OF THE AMERICAN INSTITUTE OF HOMEOPATHY

requires that no person, unless he present a diploma or certificate of graduation from an accredited university, college, academy, or high school, or a teacher's certificate, which shall be approved by the faculty as equivalent to the examinations required, shall be admitted to the second year of study, and the first course of lectures, in any of the colleges represented in this committee, without having passed a written examination upon the following subjects:

1. English composition—By writing at the time of examination, an essay of not less than 200 words, from which may be judged the writer's proficiency in grammar, spelling and writing.

2. Arithmetic—As far as square root.

3. Geography—Physical and political, as much as is contained in advanced school geographies.

4. History—Such an outline of the history of modern civilised nations, especially of the United States, as is contained in ordinary manuals of history.

5. Latin—Sufficient to read easy prose and give a fair comprehension of scientific terms and formulæ.

6. Physics—Such as is comprised in Balfour Stewart's Primer of Physics.

7. Biology and physiology—As much as is comprised in the briefer course of Martin's Human Body.

8. Chemistry — As comprised in Miller's Elementary Chemistry.

9. Botany—As found in an elementary manual.

It shall be understood that the first of these four years of study shall have been devoted to the preliminary medical studies, as outlined by this committee, and that upon successfully passing the above examination, the student shall have fulfilled the requirements of the first year of medical study."

THE NATIONAL CONFEDERATION OF ECLECTIC MEDICAL COLLEGES

demands that the preliminary requirements shall be: "1. Credible certificate of good moral standing. 2. A good English education to be attested by: (*a*) first grade teacher's certificate, or (*b*) a diploma from a graded high school, or literary or scientific college or university, or (*c*) evidence of having passed the matriculation examination to a recognised literary or scientific college, or (*d*) New York regent's medical student's certificate. 3. Also an elementary knowledge of natural history, or physics and Latin.

*Advanced Standing.*—(*a*) Graduates of reputable and regularly established colleges of dentistry, colleges of pharmacy, colleges of veterinary medicine, which require as a condition of graduation, attendance on a course extending through three or more full years, may be allowed one year's standing on a four years' medical course only on condition that they comply with the entrance requirements of the medical college, and pass all the examinations and perform all the laboratory work embraced in the course of the freshman year. (*b*) Graduates of colleges of arts and sciences which require a regular attendance of three or more years as an essential to graduation, may be admitted to the second year of the medical course without examination."

THE PHYSIO-MEDICAL COLLEGES

of Chicago, Ill., viz., Chicago Physio-Medical College and the College of Medicine and Surgery, have adopted as the preliminary educational requirements those of the Illinois State Board of Health relating to the same, which reads as follows: "1. Each applicant must present to the faculty of the college: First. Credible certificate of good moral character, signed by two physicians of good standing in the state in which the applicant last resided. Second. (1) A diploma or certificate of graduation from a high school, or (2) evidence of having passed the matriculation examination of a recognised literary or scientific college; or (3) a certificate of having successfully passed the medical student's examination conducted by the examiner, or by the faculty of a recognised literary or scientific college or university, (not members of a medical college faculty) or by the state superintendent of public instruction, or by a principal of a high school, in the following-named branches, in each of which the applicant should possess, at least, the knowledge required at the completion of one year of study in such a school, viz.: English grammar, arithmetic, elementary physics, United States history, geography and Latin. One year is allowable in which to make up defects in knowledge of Latin, but the student must be provided with a certificate of proficiency in this branch of learning from the *designated authorities* before he can be accepted as a second course student, or be given a certificate of attendance on the first year's course. Graduates of reputable and regularly established colleges of dentistry, colleges of pharmacy, colleges of veterinary medicine, which require as a condition of graduation attendance on a course extending through two or more full years, may be allowed one year's advanced standing on a four-year medical course only on condition that they comply with the entrance requirements of the medical college, and pass all the examinations and perform all the laboratory work embraced in the course of the freshman year. Graduates of colleges of arts or science which require a regular attendance of three or more years as an essential to graduation, may be admitted to second year of the medical course without examination, provided: "First, that they furnish beside their diploma or certificate of graduation, formal and satisfactory evidence of having credibly done at least one year's full work in the following-named branches, for which they may receive credit in the medical school without examination: (1) chemistry, (2) biology or comparative anatomy, (3) histology, (4) embryology, (5) experimental physics, (6) physiological botany, (7

laboratory physiology or experimental physiology, (8) zoology. And, second, that they make up their other deficiencies in the first year's work at attendance and examination, the same as other students."

THE PHYSIO-MEDICAL COLLEGE OF INDIANA

requires that an applicant desiring to enter that college furnish: "1. A certificate of good moral character, signed by two physicians of good standing in the state from which he comes. (2) The presentation of a diploma or certificate of graduation from a literary or scientific college or high school; or a certificate of successful equivalent examination by the faculty by any reputable university or college, or by the state superintendent of public instruction, or a medical student's certificate, issued by any state examining board. One year will be allowed to correct defects in the knowledge of Latin, but students must be provided with a certificate of proficiency in this subject, from designated authorities, before he can be accepted as a second course student."

THE BOARD OF REGENTS OF THE UNIVERSITY OF THE STATE OF NEW YORK

now demands as a minimum requirement the completion of satisfactory work for four years in a registered high school or its equivalent; or it grants a certificate to a student who may attain 48 counts or their equivalent by examination in the following branches: advanced English, German two years, arithmetic, rhetoric, botany, physical geography, geography, civics, economics, writing, plane geometry, Roman history, spelling, English composition, physiology, hygiene, English literature, algebra, United States history, New York history, American literature, Latin, one year, physics, first part, English history and drawing.

THE MEDICAL COUNCIL OF THE STATE OF PENNSYLVANIA

requires as a minimum standard that the applicant shall possess "the diploma of a college, diploma of an academy, seminary, normal school, or high school, or a teacher's permanent certificate, or a student's certificate of examination for admission to the freshman class in a literary college," or pass an examination in the following branches: "Arithmetic, grammar, geography, orthography, American history and English composition.

EXTENT OF THE EXAMINATION.

Arithmetic—Embracing notations, numerations, fundamental rules, multiples, factors, fractions, both common and decimal, ratio and proportion, percentage, denominate numbers, including metric system mensuration, square and cube root.

Grammar—Embracing the use of capitals, rules for punctuation, parts of speech, declensions, the formation of plurals and possessives, distinction of gender, comparison, classification and properties of verbs, elementary knowledge of the syntax of words, and analysis of easy sentences.

Geography—Including the outlines of mathematical, statistical, political, with some of the elements of physical, the political divisions, routes of commerce and travel, staple productions of the different sections of the United States.

Orthography—Such words as are commonly used in current literature.

American history—Geography of North America, the early discoverers, the character and mode of life of the natives; our forms of government from colonial times to the present, embracing the period of the Revolution, Declaration of Independence, Federal Constitution, successive administrations, with important events under each; general principles of civil government, civil war and general development. Special attention given to the history of Pennsylvania.

English composition—A general knowledge of the varieties in both prose and poetry.

Sentence—Embracing capitalisation, punctuation, grammatical classification according to form and use, words into phrases, phrases into clauses.

Paragraph—Uses of the paragraph, requisites in its construction, combination of miscellaneous sentences into paragraphs.

Prose composition—Embracing common business forms and the principal varieties of letter-writing, transformation of poetry into prose.

Theme—Its form or outline, introduction, discussion, conclusion, its kind, narrative, descriptive, persuasive, or argumentative."

THE STATE BOARD OF MEDICAL EXAMINERS OF NEW JERSEY

requires as a minimum standard that "candidates must be graduates from an accredited literary or scientific college, or have completed satisfactorily not less than a three years' course in an accredited high school or academy, or have received a preparatory education covering the following branches, viz.: orthography, arithmetic, English grammar and composition, geography, history of the United States, algebra and physics, or what the board may consider their equivalents. Applicants unable to present to the board credentials covering the above-named branches may take a special examination before the State Superintendent of Public Instructions of New Jersey, whose certificate is accepted by the board."

We might go on until we had enumerated the requirements of all the states having laws regulating the practice of medicine, and yet we would be unable to find even two of them that have exactly the same minimum requirements. It must be evident to the mind of any one, from the foregoing, that there is an urgent necessity that some uniform standard should be adopted, in justice to the medical colleges and students of the United States, and in order to enable the examining boards of the several states to perform their duties more efficiently and with fewer difficulties. Medical colleges are kept in a state of unrest and bewilderment by an endeavor to comply with the requirements of the various states governing the reception of students. Some states have laws clearly defining the minimum requirements for medical students; others have given their state boards a limited authority in making such definitions; others, again, have conferred no such power, except that which the boards may assume under the general provisions of the law. States having a minimum requirement prescribed by law cannot be expected to adopt a standard lower than that which they now enjoy, neither have the boards power to change such standards. Therefore, in order that they can join the Confederation in adopting a uniform minimum standard, it will be necessary to bring the requirements of the states having low requirements up to those having the highest, or to adopt a standard requirement, and let the states which have no power in this direction under existing laws enforce the standard adopted by the Confederation as far as they can; and endeavor by future legislation to cure the defect. It is only by such means that a uniform minimum standard can ever be adopted. It will bring no confusion to the states that cannot at once enforce such a requirement, but it will enable boards of those states to understand what is required, and to endeavor by all just methods to bring their requirements up to the standard.

Educational qualifications for entrance to medical colleges must be essentially uniform, in order that the technical courses of study builded upon them may also conform to each other. In no other way does it seem possible to establish that educational reciprocity, which is believed to be desired by all the states. A cogent reason why this body should take some definite action is that while the

Association of American Medical Colleges, the Southern Medical College Association, the Inter-Collegiate Committee of the American Institute of Homeopathy and the National Confederation of Eclectic Medical Colleges have done much to elevate and unify medical education, they do not enroll on their lists all the colleges of the United States; moreover, neither association has any legal power to enforce its requirements, whereas the National Confederation is composed of state boards, authorised and supported by law, and can therefore enforce their requirements uniformly and efficiently.

In spite of certain objections, which have been heretofore indicated, the committee has adopted as a minimum standard the four years' course in the high school. Having made this selection of a standard it is only reasonable that certain facts to substantiate the choice should be embodied in this report. The high school is found equably distributed over all the states and territories; furthermore, being maintained by public funds, it is enabled to furnish the best possible facilities and equipment free of charge to all our youth, whereas a college education can be enjoyed by few other than the more prosperous in the communities. Statistics are dry, but their argument is here convincing.

FIRST, AS TO THE AVAILABILITY OF HIGH SCHOOL ADVANTAGES ; we find that in 1897 there were 7,209 schools of this grade in the United States, having 584,900 scholars.

SECOND, AS TO THE DEMAND FOR AND DEVELOPMENT OF SUCH SCHOOLS ; the commissioner of education reports that from 1889-90 to 1896-97 the increase of students was about 219,000, being in public high schools 102 *per cent.*, and in private high schools 13 *per cent.* With the same ratio of increase a conservative estimate would place the attendance in 1899 a little short of 700,000.

THIRD, AS TO DISTRIBUTION AND ATTENDANCE ; let us consider the statistics of the public high schools in 1896-97. The states here mentioned have been taken from the North Atlantic, South Atlantic, North Central, South Central and Western divisions of the country to show that under widely differing conditions there exists a remarkable uniformity in attendance, irrespective of large centers of population, or the occupation of the people. In the United States, with a population of 71,374,142 and 7,209 high schools, the attendance was 8.19 per thousand.

| | Population. | Number of Schools. | Total Attendance. | Attendance per Thousand. |
|---|---|---|---|---|
| In New York | 6,851,000 | 548 | 57,067 | 8.33 |
| " Oregon | 378,800 | 30 | 3,015 | 7.96 |
| " Pennsylvania | 6,070,000 | 388 | 36,279 | 5.98 |
| " Texas | 2,979,000 | 261 | 17,684 | 5.93 |
| " Massachusetts | 2,634,000 | 322 | 37,641 | 14.29 |
| " Iowa | 2,101,000 | 370 | 30,698 | 14.61 |
| " Ohio | 3,834,000 | 642 | 47,415 | 12.37 |
| " Nebraska | 1,131,000 | 234 | 14,233 | 12.58 |
| " Maryland | 1,179,000 | 84 | 6,191 | 5.25 |
| " Arizona | 80,650 | 4 | 449 | 5.54 |

FOURTH, AS TO BRANCHES TAUGHT; we find a striking uniformity and a broad range in the courses of study, which include Latin, Greek, French, German, algebra, geometry, physics, chemistry, geology, physical geography, psychology, rhetoric, physiology and history. To show that these opportunities are utilised, allow me to give the number of scholars pursuing some of these branches: Latin, 248,250; German, 71,150; algebra, 280,358; geometry, 135,668; physics, 107,993; chemistry, 47,461; physical geography, 127,398; physiology, 155,000; rhetoric, 174,650; history, 186,580.

FIFTH, AS TO THE LENGTH OF COURSE; it is found that four years obtain in most of the high schools. Complete uniformity is yet to be attained, though each year shows a rapid advance toward such consummation. The National Educational Association, composed of prominent educators in our public schools, has done a grand work in its efforts to elevate the standard of our high schools, and make them uniform in scope and length of course. The aim of the association is to make the high school course a recognised unit of value in entrance qualifications to literary and scientific colleges. Thus we find another reason for adopting a standard which is in harmony with existing educational reforms; and, by such action, materially assist in their attainment. The records show that in 1897, 80,180 high-school students were pursuing courses preparatory to entering scientific and literary colleges. In this same year the graduates from high schools numbered 61,814, of whom 20,152, or 32.6 *per cent.* were prepared for college. The work of the National Educational Association is commendable to the highest degree; and, should their efforts continue as effective as they have proved for some years past, we may ere long behold the pleasing spectacle of all graduates leaving the high school fully equipped to enter the portals of the best colleges in the land.

Your committee would therefore recommend : That, as far as it be vested in the discretion of the State Examining and Licensing Boards, the rule be enforced that any college to be considered in good standing must demand as a minimum entrance requirement that the applicant possesses a high school diploma or certificate issued after a four years' course of study, or attains a satisfactory grade (75 per cent.) upon examination in the following branches, to-wit : Orthography, English grammar, English composition, geography, rhetoric, Latin, arithmetic, algebra, geometry, physics, botany, zoology and United States history. Such examination to be conducted under the direction of the state board having the authority, by certified examiners, none of whom shall be either directly or indirectly connected with a medical college.

### THE EXTENT OF THE EXAMINATION.

1. Orthography—A sufficient number of words and of such character as will be a thorough test.

2. English grammar—Embracing the parts of speech, rules of punctuation, the formation of plural and possessive, distinction of gender, classification and properties of verbs, and analysis of sentences.

3. English composition—Two compositions of not less than 200 words each. One subject to be assigned and the other subject to be elective. The compositions to be written by the student at the time of the examination. They should be criticised in relation to thought, construction, punctuation, capitalisation and handwriting.

4. Geography—Including some elements of physical geography, the political divisions, routes of commerce and travel, staple productions and population of the different countries.

5. Rhetoric—Rules and uses of rhetorical figures.

6. Latin (two years). The examination showing the ability of the student to translate and parse the construction of easy Latin prose, together with the expression in Latin of English sentences, such as would indicate two years of study.

7. Arithmetic—Such questions should be submitted as will show a clear knowledge of decimal fractions, percentage, compound numbers and square root.

8. Algebra—Through quadratics.

9. Plane geometry.

10. Physics—The questions to include the elements of mechanics, hydrostatics, hydraulics, heat, electricity and especially of optics and acoustics.

11. Botany—Embracing the structures of plants and the principles of their classification.

12. Zoology—Embracing the general divisions of animal life, with the distinctive conformations and habitat of each.

13. United States history—Boundaries and possessions of the United States, history of the early discoveries, by whom and dates, mode of life of the natives; form of government from Colonial times down to the present; various wars from the Revolution down to the present, causes of same; conditions that led to the Declaration of Independence; Federal Constitution, form of government, various administrations, dates of the most important events under each administration, growth and wealth. Candidates deficient in *one subject* may be admitted with a condition in that subject, which must be removed before the beginning of the second year.

In lieu of the foregoing credentials, medical colleges may accept (*a*) a diploma from a reputable college granting the degree of A.B., B.S., or equivalent degree; (*b*) a diploma from a normal school or seminary, legally constituted, issued after four years of study; (*c*) an official certificate of a reputable literary or scientific school, setting forth that the holder has satisfactorily pursued the studies indicated in the foregoing examination; (*d*) a teacher's permanent or life certificate; (*e*) a medical student's certificate of examination for admission to the freshman class of a reputable literary or scientific college.

One year's advance standing may be granted a student who has received the degree of A.B., B.S., or equivalent degree from a reputable literary or scientific school; provided, however, that the applicant for such standing undergoes a satisfactory examination upon every branch included in the first year's curriculum of the college to be entered. This rule to go into effect July 1, 1903.

### FORM OF CERTIFICATE ON EXAMINATION.

In order that the foregoing rule may be enforced, your committee would suggest that those having in charge the examination of students desiring to enter medical colleges be required to issue a certificate to the applicant bearing the name, age and residence of said applicant, place and date of examination, grade obtained in each branch, and signed by the person or persons holding the examination, with their official positions. This or other certificates of educational qualifications shall be presented by the student to the registrar, secretary, dean or other officer of the medical college duly authorised to admit students; and such officer shall record in a register, to be duly preserved by said college, the name, age and residence of the student, with the date, place of issuance and character of his diploma or certificate, also the date of his matriculation, and further shall record in this same register the date on which the student leaves the instructions of the college.

Upon the request of the student, the registrar, or authorised officer, shall furnish, over his signature and seal of the college, a certificate stating the name, age and residence of the student, the time of attendance and grade attained in each study pursued while a matriculant of said college. This certificate must be presented by the student to the proper officer of any college, other than the one where the original record exists and into which admission is sought, and shall be duly filed in the records of such college, together with the date of matriculation. The student may, as before, obtain from the authorised officer of this college a certificate, over the signature of said officer and seal of the college, stating time of attendance and grade obtained in each study pursued while a matriculant of said college, together with a copy of the certificate filed upon the admission of said student. In case the student is graduated, the officer authorised by the college granting the degree shall transmit to the student a certificate, over his signature and seal of the college, stating the name, age and residence of graduate, dates of matriculation in and graduation from said college, and the grades in each study pursued while a matriculant of said college, together with a copy of any certificates from other colleges relating to said graduate. This certificate, together with the diploma, credentials or preliminary educational qualifications, affidavit, and all such papers as required by law, shall be filed with the State Board of Medical Registration or Examination of that state in which the applicant desires to engage in the practice of medicine.

In closing this report your committee would earnestly appeal to the members of the Confederation for an agreement upon some definite minimum standard. While it may seem impossible to adopt any requirement conforming to each individual opinion, let us remember that our personal preferences should not be the limit to our consideration, and that we have a real duty to perform in behalf of the profession, of the state boards, and of the 150 medical colleges with their 24,000 students, found within our combined jurisdiction. The actual operation of such a requirement during a year or two would do more to fix the proper standard than a decade of theoretical discussion. We cannot afford to waste time over points of minor consideration, but let us bend our energies upon a united effort to establish a standard, and it can easily be amended thereafter, if experience demonstrates the necessity.

Respectfully submitted,

N. R. COLEMAN, Ohio, *Chairman.*
AUGUSTUS KORNDORFER, Pennsylvania.
T. J. HAPPEL, Tennessee.
GARDNER T. SWARTS, Rhode Island.
WILLIAM R. TIPTON, New Mexico.

The report was received, debated and adopted as a recommendation to the medical colleges in the United States.

